# Evolution of Allosteric Citrate Binding Sites on 6-phosphofructo-1-kinase

**DOI:** 10.1371/journal.pone.0015447

**Published:** 2010-11-23

**Authors:** Aleksandra Usenik, Matic Legiša

**Affiliations:** Department of Biotechnology, National Institute of Chemistry, Ljubljana, Slovenia; University of Queensland, Australia

## Abstract

As an important part of metabolism, metabolic flux through the glycolytic pathway is tightly regulated. The most complex control is exerted on 6-phosphofructo-1-kinase (PFK1) level; this control overrules the regulatory role of other allosteric enzymes. Among other effectors, citrate has been reported to play a vital role in the suppression of this enzyme's activity. In eukaryotes, amino acid residues forming the allosteric binding site for citrate are found both on the N- and the C-terminal region of the enzyme. These site has evolved from the phosphoenolpyruvate/ADP binding site of bacterial PFK1 due to the processes of duplication and tandem fusion of prokaryotic ancestor gene followed by the divergence of the catalytic and effector binding sites. Stricter inhibition of the PFK1 enzyme was needed during the evolution of multi-cellular organisms, and the most stringent control of PFK1 by citrate occurs in vertebrates. By substituting a single amino acid (K557R or K617A) as a component of the allosteric binding site in the C-terminal region of human muscle type PFK-M with a residue found in the corresponding site of a fungal enzyme, the inhibitory effect of citrate was attenuated. Moreover, the proteins carrying these single mutations enabled growth of *E. coli* transformants encoding mutated human PFK-M in a glucose-containing medium that did not support the growth of *E. coli* transformed with native human PFK-M. Substitution of another residue at the citrate-binding site (D591V) of human PFK-M resulted in the complete loss of activity. Detailed analyses revealed that the mutated PFK-M subunits formed dimers but were unable to associate into the active tetrameric holoenzyme. These results suggest that stricter control over glycolytic flux developed in metazoans, whose somatic cells are largely characterized by slow proliferation.

## Introduction

The ATP-dependent enzyme 6-phosphofructo-1-kinase (PFK1, EC 2.7.1.11) catalyzes one of the three irreversible steps of glycolysis, a process that is central to primary metabolism. It catalyzes the Mg-ATP-dependent phosphorylation of fructose-6-phosphate (F6P), resulting in its conversion to fructose 1,6-bisphosphate (F1,6P) and the release of Mg-ADP as a byproduct [1]. The enzyme is present in bacteria, fungi and animals, whereas in plants another type of 6-phosphofructo-1-kinase (EC 2.7.1.90) is predominant, which uses pyrophosphate as a phosphoryl donor [2]. PFK1 is the site of the most complex control over the glycolytic flux, and allosteric regulation is one of the strategies used to control catalysis.

Sequence analyses of prokaryotic and eukaryotic ATP-dependent PFK1 enzymes suggest that they diverged via duplication and tandem fusion of a prokaryotic ancestor gene [3].Eukaryotic PFK1s are therefore more than twice the size of prokaryotic PFK1s and are under regulatory control by a wider array of effectors than the simpler bacterial enzymes. A total of six organic ligand binding sites are found in eukaryotic enzymes: the catalytic ATP and F6P binding sites, activator-binding sites for adenine nucleotides and fructose-2,6-bisphosphate (F2,6P) and inhibitor–binding sites for ATP and citrate [3], [4]. However, the strict conservation between the active site residues in the N-terminal half of the eukaryotic enzyme and those of bacterial PFK1s suggests that the only active site in the eukaryotic enzyme is located in the N-terminus [3]. On the other hand, the allosteric ligand binding sites that developed because of mutations in the C-terminal region enable fine-tuning of the regulatory enzyme in response to elevated levels of specific downstream metabolites.

One of these allosteric modulators is citrate. Studies on allosteric citrate binding site in rabbit muscle PFK1 concluded that it developed from the phosphoenolpyruvate (PEP)/ADP binding site of the prokaryotic PFK1s. Amino acid residues involved in the citrate binding are therefore found both on the N- and C-terminal part of the molecule and were determined by single point mutations [5], [6] or by chemical modification [7]. So far, crystal structures of the ATP-dependent PFK1s from two prokaryotic microorganisms have been determined; *E. coli* [8] and *L. bulgaricus* [9]. Only one structure of PFK1 from a eukaryote (*Trypanosoma brucei*) was described [10], however this protozoan enzyme has not been subjected to gene duplication/fusion event that is characteristic for other eukaryots. Unfortunately, until now the mammalian PFKs have proved recalcitrant to crystallization and subsequent X-ray analysis. Therefore, only models for mammalian PFKs were constructed. From a proposed model for the evolution of the ligand biding sites prepared by Gunasekera and Kemp [6], the binding site for citrate that is located between the N- and C- terminal region, might form a gap between both parts of the enzyme. However, there is no information about the actual mechanism of interaction between the specific residues and citrate, nor about the number of citrate molecules needed for inhibition.

Citrate seems to play a major role in downregulation of PFK1 and therefore glycolytic flux. In fact, citric acid build-up is a sign of anaplerotic conditions in the cells, which are characterized by elevated levels of tricarboxylic acid (TCA) cycle intermediates and by the need to slow down glycolysis. It is generally known that citrate does not affect bacterial PFK1; however, some inhibition by citrate can be found in PFK1 enzymes from eukaryotic microbial species. In the filamentous fungus *Aspergillus niger,* enzyme activity was inhibited by 50% by doses of citrate ranging from 4 to 6 mM [11], [12]. When the effect of citrate on the activities of PFK1 isoforms expressed in nervous and muscular tissues from various species was studied, strong inhibition by citrate was found for vertebrate species, with weaker inhibition seen in insects [13]. More detailed studies on rat PFK1 isoforms revealed that the enzymes' activities were halved at 0.08, 0.13 and 0.18 mM of citrate for platelet (PFK-P), muscle (PFK-M) and liver (PFK-L) enzymes, respectively [14]. That glycolysis is highly controlled at the PFK1 step was also confirmed by calculations of enzymatic flux capacities (V_max_) and maximum physiological flux rates (v) in animal muscle. Whereas flux capacities far exceed physiological velocities in low-flux muscles, in high-flux muscles a close match between flux capacities and flux rates is observed. However, PFK1, in contrast to hexokinase and glycogen phosphorylase, does not function at a velocity close to V_max_ in exercising muscle, suggesting a complex role for this key glycolytic enzyme. Again, this effect seems to be more evident in vertebrates than in insects [15]. It is therefore tempting to speculate that during the evolution of metazoans more strict control over glycolytic flux might have been required. Such control may have emerged through the selection of specific mutations enabling more strict control of PFK1 activities by one of its downstream products, the TCA cycle intermediate citric acid.

In the present paper, we present evidence showing that single amino acid residues at the specific citrate binding sites can determine the sensitivity of the enzyme toward this TCA cycle metabolite. During the evolution of metazoans, the selection of these mutations resulted in stronger inhibition of PFK1 by citrate, suggesting the importance of strict control over glycolytic flux in mammalian cells.

## Material and Methods

### DNA manipulations

DNA manipulations were essentially done as described by Sambrook and Russel [16]. PCR reactions were performed with Platinum® *Pfx* DNA polymerase (Invitrogen, Carlsbad, CA) using the reaction solution recommended by the manufacturer. DNA was sequenced by Eurofins MWG Operon (Ebersberg, Germany).

Human muscle type 6-phosphofructo-1-kinase cDNA (Clone ID 2964710) was purchased from Geneservice Ltd. (www.Geneservice.co.uk). The native human *pfk*-M gene was amplified by PCR using 5′-AATTATGGATCCATGACCCATGAAGAGCACC-3′ as a forward primer and 5′-AATTATTCTAGATTAGACGGCCGCTTCCCC-3′ as a reverse primer. At the same time, restriction sites were introduced at the 5′ (*Bam*HI) and 3′ (*Xba*I) ends that enabled cloning into the pALTER-Ex1 plasmid (Promega, Southampton, UK) under the control of the *tac* promoter. Mutations (K557R, D591V, K617A, D591V/K617A, K557R/D591V/K617A) were generated in the native human *pfk*-M inserted into pALTER-Ex1 by the use of QuikChange® II XL Site-Directed Mutagenesis Kit (Stratagene, La Jolla, CA). For the introduction of the K557R, D591V and K617A mutations the following forward mutagenic primers were used: 5′-CCTGTGACCGCATCAGGCAGTCAGCAGCTGG-3′, 5′-GGACTGGCAGCTGGGGCCGTCGCTGCCTACATTTTTGAG-3′ and 5′-GAACATCTGGTGCAAAAGATGGCCACAACTGTGAAAAGGGGCTTG-3′; for reverse primers, the complementary reverse oligonucleotides were used. All primers were synthesized by Eurofins MWG Operon (Ebersberg, Germany). Finally, the correct nucleotide sequences of the native gene and all mutated genes were verified.

### Expression and purification of recombinant enzymes

Plasmids were initially propagated in the *E. coli* strain JM109 (Promega, Southampton, UK) by growing the bacteria in LB medium with tetracycline (10 µL/mL). Native and mutant forms of human *pfk*-M gene were subsequently expressed in the *pfk* double-knockout *E. coli* strain RL257 (F-, [araD139]_B/r_, lacIp-4000(lacI^Q^), e14-, pfkB205(del-ins)::FRT, flhD5301, Δ(fruK-yeiR)725(fruA25), relA1, rpsL150(strR), rbsR22, pfkA203(del-ins)::FRT, Δ(fimB-fimE)632(::IS1), deoC1) [17]. *E. coli* RL257 transformants were grown in 1 liter of LB medium with tetracycline on a rotary shaker at 37°C until an OD_600_ of 0.5 was reached. Isopropylthiogalactoside (IPTG) was added to a final concentration of 1 mM, and the incubation continued for 16 h at 30°C. The cells were harvested by centrifugation at 5,000× g for 10 min, washed with 50 mL of ice-cold extraction buffer (100 mM sodium phosphate buffer (pH 7.8), 0.15 M glycerol, 1 mM DTE, 1mM PMSF, 1 mM EDTA). The precipitate was frozen under liquid nitrogen and stored at −80°C until needed.

Frozen bacterial cells were disrupted in a Mikro-Dismembrator (Sartorius AG, Gottingen, Germany). Cell free homogenate was extracted with 10 mL of cold 100 mM sodium phosphate buffer (pH 7.8) containing 0.15 M glycerol, 1 mM DTE, 1mM PMSF, 1 mM EDTA and 10 µL/mL of protease inhibitor cocktail (Sigma-Aldrich, Steinheim, Germany). The same buffer was used throughout the whole isolation procedure. Dissolved proteins in the supernatant formed after centrifugation at 16,000× g for 20 min at 4°C and were precipitated with ammonium sulfate, and a fraction of between 45 and 75% of saturation was taken for further purification. After dissolving precipitated proteins and desalting the sample on a Sephadex ^TM^ G-25 column (GE Health Care, Piscataway, NJ), the proteins were loaded onto an affinity column containing 1 mL of aminophenyl-ATP-Sepharose (Jena Bioscience, Jena, Germany) that had been previously equilibrated with extraction buffer. After the sample was applied to the column, unbound proteins were removed by extensive washing. The PFK-M enzyme was eluted from the column with 1.5 mL of buffer containing 6 mM F6P and 1 mM ADP. Eluted enzyme was dialyzed overnight against a buffer containing 20% (v/v) glycerol and stored at 4°C.

### Testing transformants for growth in glucose-containing medium

Transformed *E. coli* RL257 cells were grown overnight in 10 mL of LB medium with tetracycline (10 µL/mL) at 37°C. One hundred milliliters of M63 minimal medium with glucose (10% w/v), tetracycline (10 µL/mL) and 0.8 mM IPTG was inoculated with an adequate volume of the overnight *E. coli* culture. During subsequent growth at 30°C, aliquots of the culture were removed at the indicated times, and the optical density was measured at 600 nm.

### Enzyme assays

PFK1 activity was measured spectrophotometrically at 340 nm (Lambda25 UV/VIS spectrophotometer, Perkin Elmer) essentially as reported previously [18] using a coupled reaction system. Unless otherwise stated, the assay mixture contained, in a final volume of 1 mL: 50 mM HEPES buffer (pH 7.8), 1 mM DTE, 100 mM KCl, 5 mM MgCl_2_, 0.2 mM NADH, 0.025–2 mM F6P, 0.9 U/mL aldolase (Sigma-Aldrich, Steinheim, Germany), 15 U/mL triosephosphate isomerase and 15 U/mL glycerol-3-phosphate dehydrogenase (Sigma-Aldrich, Steinheim, Germany). Before use, the auxiliary enzymes were dialyzed overnight at 4°C against 50 mM HEPES buffer (pH 7.8) containing 1 mM DTE, with one change of buffer after 8 h. Different concentrations of citrate were added to the assay mixture just before the reaction was started by the addition of ATP to a final concentration of 0.5 mM. Concentration of the enzyme used in the kinetic assays was 0,2 µg/mL. All presented kinetic data are averages obtained from a minimum of three replicate measurements. Total protein concentrations of the samples were determined using a Bio-Rad protein assay (Bio-Rad, Hercules, CA) with bovine γ-globulin as a standard [19].

### Immunoblotting

Aliquots of supernatants prepared from cell-free homogenates of *E. coli* transformants were applied to a Sepharose 12 column connected to a FPLC system (Pharmacia, Uppsala, Sweden). Appropriate fractions were collected after chromatography. Calibration curves for the determination of protein molecular masses in individual fractions were obtained using both high (HMW) and low molecular weight (LMW) gel filtration calibration kits (Pharmacia, Uppsala, Sweden). Fractions of dissolved proteins collected after chromatography were separated using SDS-PAGE with 10% polyacrylamide gels and 0.1% sodium dodecyl sulphate and transferred onto nitrocellulose membranes. The membranes were blocked with I-Block (Tropix Inc., Bedford, MA), washed and incubated with 1∶1000 dilutions of purified primary antibodies. Polyclonal rabbit antibodies were raised against an epitope specific for human PFK-M (CKDFREREGRLRAA; GenScript Corporation, www.genscript.com). The membranes were then rinsed with 1∶2000 dilutions of goat anti-rabbit-HRP secondary antibodies (Abcam, Cambridge, UK). The blots were developed with ECL detection reagents (Amersham, GE Healthcare) and the membrane exposed to BioMax^™^ XAR film (Eastman Kodak, Rochester, NY). The film was developed using an Ilford PQ Universal paper developer (Harman Technology Ltd., Mobberley, UK).

## Results

### Citrate binding sites in various organisms

**Figure 1:**
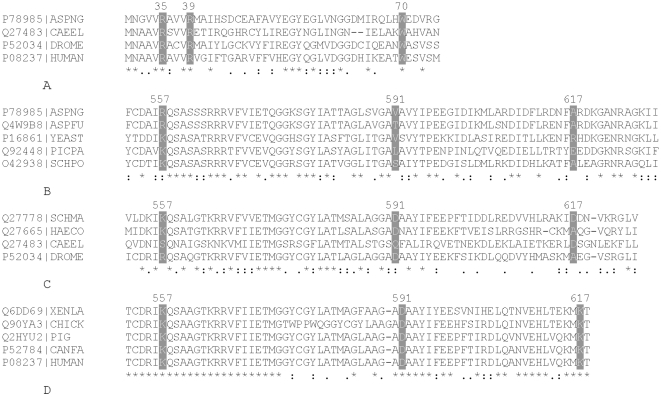
Multiple sequence alignment of amino acid residues at the N and C-termini of PFK1 proteins that form allosteric citrate binding sites. A. Amino acid residues (grey background) of citrate binding sites in the N-terminal region of PFK1 isoforms from the following species: ECO24, *Escherichia coli*; ASPNG *Aspergillus niger*; CAEEL *Caenorhabditis elegans;* DROME, *Drosophila melanogaster*; HUMAN, *Homo sapiens*. B. Amino acid residues (grey background) of citrate binding sites in the C-terminal region of PFK1 isoforms from the following fungi: ASPNG, *Aspergillus niger*; ASPFU, *Aspergillus flavus*; Yeast, *Saccharomyces cerevisiae*; PICPA, *Pichia pastoris*; SCHPO, *Schizosaccharomyces pombe*. C. Amino acid residues (grey background) of citrate binding sites in the C-terminal region of PFK1 isoforms from the following invertebrates: SCHMA, *Schistosoma mansoni*; HAECO, *Haemonchus contortus*; CAEEL, *Caenorhabditis elegans;* DROME, *Drosophila melanogaster*. D. Amino acid residues (grey background) of citrate binding sites in the C-terminal region of PFK1 isoforms from the following species: XENLA, *Xenopus laevis*; CHICK, *Gallus gallus*; PIG *Sus scrofa*; CANFA, *Canis familiaris*; HUMAN, (*Homo sapiens*). The components of allosteric citrate site were originally identified in the mouse PFK-C enzyme (Accession number Q9WUA3) [6], which has 69,58% of identical; 13,18% strongly similar and 6,46% weakly similar residues to the human PFK-M (Accession number P08237); however, there is a minor shift in numbering of amino acid residues between the enzymes. The mouse PFK-C enzyme has an extension of 8 amino acid residues at the N-terminal end of the enzyme and an insertion at position 349. Therefore, the corresponding ligand binding sites in the N-terminal part of human PFK-M differ by 8 amino acid residues and in the C-terminal region by 9 residues with respect to the mouse PFK-C. The numbering system for amino acids used in the entire paper, therefore reflects the positions on the human PFK-M. The alignments were generated using CLUSTAL W [34].

Analyses of citrate binding sites on the PFK1 enzymes of various eukaryotic organisms revealed that identical amino acid residues were found at the N-terminus in all examined species (Fig. 1A); however, more variance was observed among the binding sites at the C-terminus. Position 557 in the human PFK-M and corresponding positions in the enzymes from other organisms was typically occupied by lysine (K) or arginine (R) residues (Fig. 1B–D). Position 600 in fungal enzymes, that corresponded to position 591 in human PFK-M was typically ocupied by non-ionizable amino acid residues with either polar or hydrophobic properties (Fig. 1B), but in lower animals the ionizable acidic residue aspartic acid was normally present (Fig. 1C). At the last position, corresponding to position 617 of human PFK-M, hydrophobic residues were predominant in lower eukaryotes (Fig. 1B), but either acidic residues such as aspartic acid (D) or else the hydrophobic amino acid alanine (A) were found in invertebrates (Fig. 1C). On the other hand, lysine (K) was only present in vertebrates (Fig. 1D). These data suggest that specific C-terminal residues alone may determine the sensitivity of the enzyme to inhibition by citrate. Strict conservation of individual components of citrate binding sites throughout vertebrate species, where PFK1 enzymes are reported to be highly sensitive to citrate inhibition, implies that this specific pattern of residues must have been selected for during the evolution of higher animals. To verify this hypothesis, specific residues in the human PFK-M were replaced with the corresponding residues from the fungal *A. niger* PFK1. Properties of the single mutants K557R, D591V and K617A, a double mutant, D591V/K617A, and a triple mutant, K557R/D591V/K617A, were evaluated.

**Figure 2:**
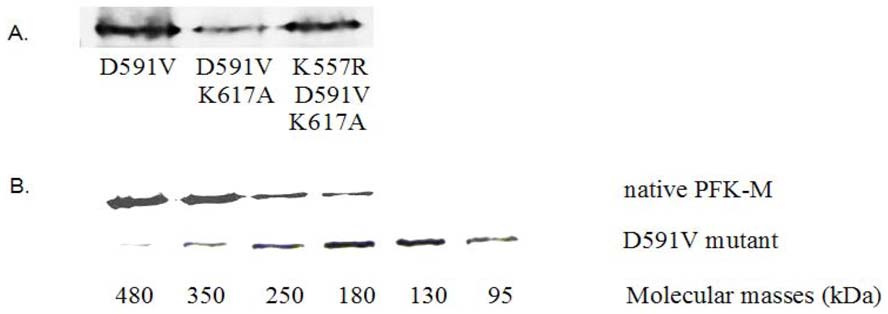
Western blot analyses of *E. coli* transformants. **A**. Western blots of inactive mutant forms of human PFK-M synthesized in *E. coli* transformants grown in LB medium. **B**. Western blots of specific fractions collected after gel filtration of homogenate prepared from *E. coli* transformants encoding wild type human PFK-M (above) and its inactive mutant D591V (below). Molecular weights of the proteins in individual fractions as determined using the calibration curve are shown in the bottom line.

*E. coli* strain RL257, which lacks its own *pfk* genes, was used for transformation of the native PFK-M cDNA and mutated genes. Although no PFK1 activity of the mutated PFK1 enzymes D591V, D591V/K617A and K557R/D591V/K617A could be detected in cell homogenates of *E. coli* transformants grown on LB medium, the expression of inserted genes was confirmed by western blotting (Fig. 2A).

### Kinetic properties of the native and mutant forms of human PFK-M

**Figure 3:**
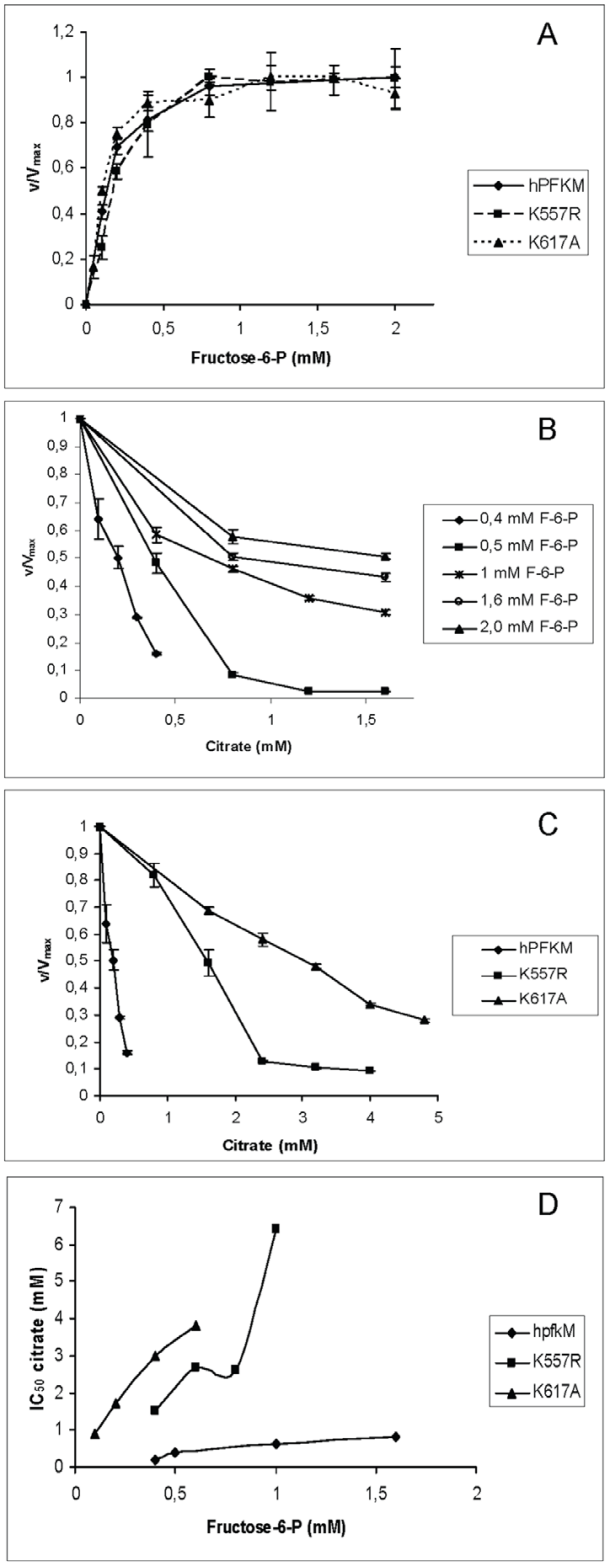
Kinetic measurements of recombinant human PFK-M and mutant forms of PFK-M. **A. Fructose-6-phosphate (F6P) saturation curves for the native human and mutant forms of PFK-M.** Measurements were carried out at pH 7.8 in a buffer containing 5 mM Mg^2+^ and 0.5 mM ATP. Activities are expressed as a ratio of enzyme activity (v) at a specific substrate concentration to the activity detected at saturating F6P concentration (V_max_). Data are presented as means ± standard deviation. **B. Citrate inhibition of the native human PFK-M measured at different fructose-6-phosphate (F6P) concentrations.** The assay was performed at pH 7.8 in a buffer containing 5 mM Mg^2+^ and 0.5 mM ATP. Data are presented as means ± standard deviation. **C. Citrate inhibition of the native and mutant forms of human PFK-M.** All measurements were conducted at 0.4 mM F6P. The assay was carried out at pH 7.8 in the presence of 5 mM Mg^2+^ and 0.5 mM ATP. Activities are expressed as a ratio of activity detected in the presence of citrate to activity measured without citrate in the system. Data are presented as means ± standard deviation. **D. IC_50_ values for citrate inhibition of the native and mutant forms of human PFK-M measured at increasing concentrations of F6P.** The assay was carried out at pH 7.8 in the presence of 5 mM Mg^2+^ and 0.5 mM ATP. Data were obtained by determining the citrate concentration that caused inhibition of the wild type and mutated forms of PFK-M by 50%. Mean values of at least three independent measurements are reported.

The activities of isolated native human PFK-M and two mutants (K557R and K617A) were measured at increasing concentrations of F6P in the presence of 5 mM Mg^2+^ and 0.5 mM ATP at pH 7.8. All enzymes showed maximum activities in the range of 110–200 units/mg of protein, and these activities were reached at 1 mM F6P. The K_m_ values of the native enzyme and both mutants were roughly identical and were all around 0.1 mM (Fig. 3A). These results show that these mutations had no effect on the basic kinetics of the PFK-M enzyme.

#### Citrate inhibition of the native and mutant forms of human PFK-M

To assess the inhibitory effect of citrate on the various PFK-M isoforms, citrate in the form of its tri-potassium salt was added to the measuring system. Increasing concentrations of citrate led to the gradual inhibition of the native enzyme; however, the inhibitory effect was diminished at higher concentrations of F6P. While the IC_50_ for citrate was 0.2 mM in the presence of 0.4 mM of substrate, 0.6 mM of citrate was needed to reduce the enzyme's activity by half at 1 mM F6P. When F6P concentrations in the system were greater than 1.5 mM, the enzyme showed sensitivity to citrate only at concentrations below 1 mM, whereas higher concentrations of the inhibitor had no additional negative effect on the enzyme's activity (Fig. 3B). The K_i_ value for citrate acting on the native human enzyme was calculated to be 0.05 mM. As presented in Figure 3C, the K557R and K617A amino acid substitutions markedly decreased the inhibitory effect of citrate. The K557R mutant was shown to be more sensitive to citrate inhibition than the K617A mutant; they exhibited K_i_ values of 0.3 mM and 0.5 mM, respectively. As for the native enzyme, a decreased inhibitory role of citrate was observed at increasing substrate concentrations for both mutants (Fig. 3D).

### Growth of *E. coli* transformants encoding the native human PFK-M enzyme and two mutated PFK-M enzymes in glucose-containing media

**Figure 4:**
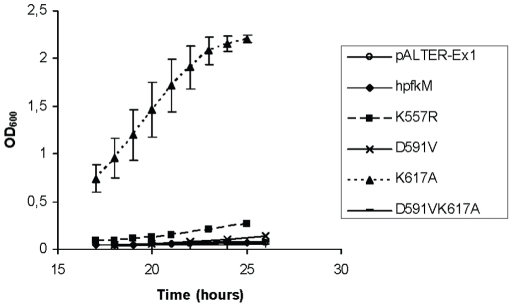
Growth of *E. coli* RL 257 transformants encoding human native and mutant PFK-M forms. Growth was recorded in a minimal medium with glucose as the sole carbon source at 30°C. Data are presented as means ± standard deviation.

All transformants were also tested for growth on glucose. An *E. coli* RL257 strain transformed with pALTER-Ex1 plasmid without any insertions was used as a negative control. It is worth noting that the parental *E. coli* strain RL257 (with no active PFK enzyme) was unable to grow on minimal medium with glucose. The transformants were tested for growth on M63 minimal medium including 10% (w/v) of glucose. As expected, transformants encoding the mutants D591V, D591V/K617A and K557R/D591V/K617A showed no growth over the observed time period. Previously, no PFK-M activity in these transformants grown on complex medium was detected. Surprisingly, no growth was observed with the transformant encoding the native human PFK-M, whereas a modest growth reaching an OD_600_ value of 0.26 after 25 h was detected with the K557R mutant. The only transformant that showed a significant growth rate (with an approximate doubling time of 3 h) was the one encoding the K617A mutant (Fig. 4).

Since no PFK1 activities and no growth of the transformants carrying the proteins with D591V substitution was observed, we speculated that this mutation inactivated human PFK-M and that the other two mutations, K557R and K617A, might not compensate for the loss of PFK1 activity. Since the D591V mutation might prevent the consolidation of monomers into an active tetrameric structure, the quaternary structures of the enzymes were assessed by running cell free homogenate through a size-exclusion column and detecting PFK-M monomers by western blot in different eluted fractions. Tests were performed on the native human PFK-M and the D591V mutant. For the homogenate of the native PFK-M transformant, the strongest western blot signals were recorded in fractions 10.5 and 11 mL, which corresponded to proteins of molecular masses of around 350 kDa. In contrast, the strongest western blot bands in the homogenate of the D591V transformant were detected in fractions 11.5 and 12 mL, where proteins with molecular masses of 170 kDa were predominant (Fig. 2B). The reported molecular mass of a single human PFK-M monomer is 85,183 Da [20]. These data suggested that the substitution of the aspartic acid (D) residue at position 591 with valine (V) enabled binding of two monomers into a dimer but prevented the formation of an active tetrameric PFK-M structure. It should be reminded that the mutations on human PFK-M enzyme were carried out by replacing corresponding residues from the fungal *A. niger* PFK1. Since the *A. niger* enzyme was active despite of valine present at position 591, arginine at position 557 and alanine at position 617, other regions in the C-terminal part must be involved in the formation of a quaternary structure of the holoenzyme.

## Discussion

The catabolic reactions in cells encompassing glycolysis, tricarboxylic acid cycle and oxidative phosphorylation are often termed “primary metabolism”. Although an important feature of catabolism is the generation of chemically conserved energy in the form of ATP, numerous intermediates of primary metabolism act as precursors for the formation of cellular building blocks that enable cell growth and division. Whereas in the microbial world the overall growth rate is normally controlled by the availability of nutrients from the environment, in metazoans nutrient levels in the vascular system do not vary much. In order to control the proliferation of somatic animal cells, more strict regulation of primary metabolism must have been developed during evolution. An important point of control over the metabolic flux of primary metabolism seems to be the enzyme PFK1.

Analyses of the allosteric citrate binding sites and kinetic characteristics of eukaryotic PFK1 enzymes revealed that stronger inhibition by citrate has been selected for during the development of metazoans. The most powerful regulatory effect of citrate as a feedback inhibitor was recorded in *Vertebrata*, whose PFK1 isoforms have conserved amino acid residues forming the citrate allosteric sites at both the N and C-terminal regions. Amino acid motifs responsible for citrate binding at the C-terminus are characterized by two basic residues and one acidic residue that apparently enable strong allosteric effects of the ligand on the protein. In contrast, in fungi, where less stringent control over glycolytic flux is required, only one component of allosteric site in the C-terminal part is of this basic-ionizable type while the other two are predominantly non-ionizable (some are even hydrophobic). Similarly, in lower animals (invertebrates) basic residues such as lysine and arginine are found at one position, while the next two components are characterized predominantly by the presence of ionizable-acidic residues.

Interestingly, a single substitution of valine (V) for aspartic acid (D) at position 591 resulted in loss of activity. As revealed by gel filtration, monomers containing this mutation were unable to form tetrameric structures and remained dimeric. Although amino acid residues enabling association of monomers into a dimer have been suggested [21], further studies of the residues at position 591 and in the surrounding area might reveal grouping of dimers into active tetrameric structures.

The mechanism of citrate interaction with individual components of citrate allosteric site on PFK1 enzymes has not been studied yet on a submolecular level. However, the importance of specific amino acid residues at allosteric citrate interaction site in mammalian PFK-M was demonstrated by replacing a basic residue at position 617 with a hydrophobic one. This single substitution diminished the enzyme's sensitivity to citrate. Moreover, it enabled the recombinant enzyme to participate actively in bacterial metabolism, which was reflected by the growth of transformants in a glucose-containing medium. It is important to realize that the native human PFK-M enzyme didn't enable growth of *E. coli* RL257 on glucose, although its activity was detected in a cell free homogenate. This might be due to the high sensitivity of mammalian PFK-M enzymes to citrate inhibition. The estimated intracellular citrate concentration during the exponential growth phase on glucose was reported to be approximately 0.9 µmol/g cell dry weight in *E. coli* [22], which equals approximately 400 µM if the cellular volume is assumed to be 2.3 mL/g of dry weight [23]. The possible role of intracellular citrate concentration on growth of transformants carrying human PFK-M enzymes was further shown by both PFK-M mutants. K557R mutant carrying PFK-M enzyme with IC_50_ value of 0,3 mM enabled slow growth of transformant, while K617A mutant with PFK-M enzyme less sensitive to citrate inhibition (IC_50_ = 0,5 mM) grew faster.

A correlation between PFK1 activity and cell proliferation rate has been observed also in cancer cells. By inhibiting Fructose-2,6-bisphosphate formation, a potent activator of eukaryotic PFK1 enzymes, markedly attenuated proliferation of several tumorigenic cell lines was observed [24].

Data regarding accurate cytosolic citrate concentrations in eukaryotes would be very informative. Relative high concentrations were reported for fungal cells; in *Aspergillus niger* the citrate concentration was between 2 mM and 30 mM [25] and in *Saccharomyces cerevisiae* it was between 2.4 mM [26] and 3.5 mM [27]. In normal human tissues, the measured citrate levels were in the range of 200–450 µM [28]. However, these values do not take into consideration the compartmentalization of citrate into mitochondria and cytosol. It is worth noting that citrate is formed in the mitochondrial matrix, while PFK1 is strictly cytosolic. However, a portion of the mitochondrial citrate is regularly transferred into the cytosol and used for lipid acid synthesis after being converted to acetyl-CoA by ATP-citrate lyase [29].

Weaker inhibition of PFK1 by citrate was observed at higher concentrations of F6P. This effect appears to be of moderate physiological relevance, since intracellular concentrations of F6P are relatively low. The intracellular concentration of F6P is reported to be 0.2 mM in *A. niger* [18], 0.23 mM in *S. cerevisiae* [27] and 0.08–0.17 mM in mammalian skeletal muscle [30].

Feedback inhibition of PFK1 and concomitant regulation of metabolic flux through the glycolytic pathway obviously fails in mammalian tumor cells. In malignant tissues, citrate concentrations that are significantly higher than normal (reaching values of 200–2000 µM) have been reported [27]. Dysregulation of glycolysis, also known as the Warburg effect, is characteristic of cancer cells [31]. There have been several reports of PFK1 isoforms with atypical kinetic characteristics in tumors. A PFK1 isoform with reduced sensitivity to citrate inhibition (Ki = 0.75 mM citrate) and increased sensitivity to activation by F2,6P was described in human glioma cells (as compared to the enzyme from normal brain tissue (K_i_ = 0.1 mM citrate); [32]. A PFK1 enzyme with similar kinetic characteristics was observed in the fast growing rodent hepatoma cell line AS-30D; this form of PFK1 showed complete insensitivity toward its allosteric inhibitors citrate and ATP in the presence of physiological concentrations of F2,6P. On the other hand, the enzyme was highly activated by its activators NH_4_^+^, AMP and F2,6P [33]. However, the nature of PFK1 isoforms exhibiting changes in enzyme kinetics has not been studied in detail.

In conclusion, these results indicate that amino acid residues of allosteric binding site for citrate at the C-terminus of PFK1 enzymes determine the strength of inhibition by citrate. By substituting a specific amino acid residue, the level of inhibition of the enzyme can be modulated. Analyses of the variations in allosteric binding sites among different eukaryotic organisms revealed that stronger inhibition of PFK1 enzymes by citrate has developed during evolution, enabling better control over glucose consumption in the slowly growing somatic cells of higher metazoans. On the other hand, no downregulation of PFK1 isoforms by feedback inhibition has been described in cancer tissues, which are characterized by rapid cell growth and proliferation.
